# Crania Ægyptiaca; or Observations on Egyptian Ethnography, Derived from Anatomy, History, and the Monuments

**Published:** 1844-07-01

**Authors:** 


CRANIA ÆGYPTIACA; OR OBSERVATIONS ON EGYPTIAN ETHNOGRAPHY, DERIVED FROM ANATOMY, HISTORY, AND THE MONUMENTS. By *Samuel George Morton*, M.D. Author of Crania Americana, &c. &c. Philadelphia, 1844. London, Madden and Co.

OUR readers will remember that, in a former number of this Journal, we gave some account of Dr. Morton's Researches on American Ethnography. He has lately taken a bolder flight, and explored the dark chambers of the Catacombs, the Pyramids, and the mouldering monuments of that Cradle of the Arts, that Parent of Civilization, that Land of Mystery—EGYPT. Were it not for the sepulchres of a country whose history confounds chronology, the very people who thronged her cities would be unknown to us. But those numberless vaults have given up their dead for ages past, and will for ages to come, to bear witness for themselves and their country. Little did the kings, queens, and magnates of Egypt believe or suspect that their carefully embalmed bodies would be sacreligiously unrolled and unswathed, thousands of years after sepulture, to gratify the curiosity of antiquarians, and embellish the walls of museums in countries of which they never heard even the names!

Notwithstanding the innumerable resurrections of mummies from their receptacles on the banks of the Nile, yet still the physical characteristics of the Ancient Egyptians are regarded with great diversity of opinion by the learned antiquarians, who variously refer them to the Jews, Arabs, Hindoos, Nubians, and even the Negroes. The details of organic structure have been involved in the same uncertainty—and the great problem is still unsolved—did Civilization ascend or descend the Nile? Did Egypt or Œthiopia give it origin? Our author was fortunate in the friendship and the industry of G. R. Gliddon, Esq. who resided long in Egypt, as Consul for the United States, and who, in the course of his travels, both in Egypt and Nubia, procured 137 human crania, of which 100 pertain to the ancient inhabitants of Egypt. Of these last, seventeen were sent to Dr. Morton through the medium of Clot Bey—who arranged them in two series, the Pharaonic and the Ptolemaic. The object of this, our author's memoir is, therefore, to throw some additional light on the questions above stated, and to ascertain, if possible, the Ethnographic characters of the primitive Egyptians—or, in other words, to point out their relative position among the races of men.

This investigation is carried on with most laborious industry, and illustrated by numerous—we had almost said innumerable, plates and woodcuts, without the aid of which, the reasonings and arguments could not be understood. This memoir is so carefully worded—so terse and concentrated in itself—so constantly elucidated by the plates, that it is totally incapable of analysis or compression. Neither can we, by a series of extracts, convey anything like an idea of this vast investigation. One only extract can we make, shewing something like the plan of the curious and highly interesting volume before us.

### Egyptian Ethnography.

" It was remarked fifty years ago by the learned professor Blumenbach, that a principal requisite for an inquiry such as we now propose, would be 'a very careful technical examination of the skulls of mummies hitherto met with, together with an accurate comparison of these skulls with the monuments.' This is precisely the design I have in view in the following memoir, which I therefore commence by an analysis of the characters of all the crania now in my possession. These may be referred to two of the great races of men, the Caucasian and the Negro, although there is a remarkable disparity in the number of each. The Caucasian heads also vary so much among themselves as to present several different types of this race, which may, perhaps, be appropriately grouped under the following designations:—

### CAUCASIAN RACE.

" 1. The *Pelasgic Type*. In this division I place those heads which present the finest conformation, as seen in the Caucasian nations of western Asia, and middle and southern Europe. The Pelasgic lineaments are familiar to us in the beautiful models of Grecian art, which are remarkable for the volume of the head in comparison with that of the face, the large facial angle, and the symmetry and delicacy of the whole osteological structure.

" 2. The *Semitic Type*, as seen in the Hebrew communities, is marked by a comparatively receding forehead, long, arched, and very prominent nose, a marked distance between the eyes, a low heavy broad, and strong and often harsh development of the whole facial structure.

" 3. The *Egyptian* form differs from the Pelasgic in having a narrower and more receding forehead, while the face being more prominent, the facial angle is consequently less. The nose is straight or aquiline, the face angular, the features often sharp, and the hair uniformly long, soft, and curling. In this series of crania I include many of which the conformation is not appreciably different from that of the Arab and Hindoo; but I have not, as a rule, attempted to note these distinctions, although they are so marked as to have induced me, in the early stage of the investigation, and for reasons which will appear in the sequel, to group them, together with the proper Egyptian form, under the provisional name of *Austral-Egyptian* crania. I now, however, propose to restrict the latter term to those Caucasian communities which inhabited the Nilotic valley *above* Egypt. Among the Caucasian crania are some which appear to blend the Egyptian and Pelasgic characters: these might be called *Egypto-Pelasgic* heads; but without making use of this term, except in a very few instances by way of illustration, I have thought best to transfer these examples from the Pelasgic group to the Egyptian, inasmuch as they so far conform to the latter series as to be identified without difficulty.

### NEGRO RACE.

" The true *Negro* conformation requires no comment; but it is necessary to observe that a practised eye readily detects a few heads with decidedly mixed characters, in which those of the Negro predominate. For these I propose the name of *Negroid* crania; for while the osteological development is more or less that of the Negro, the hair is long but sometimes harsh, thus indicating that combination of features which is familiar in the mulatto grades of the present day. It is proper, however, to remark in relation to the whole series of crania, that while the greater part is readily referrible to some one of the above subdivisions, there remain other examples in which the Caucasian traits predominate, but are partially blended with those of the Negro, which last modify both the structure and expression of the head and face.

"We proceed, in the next place, to analyze these crania individually, arranging them, for the purpose of convenience, into seven series, according to their sepulchral localities, beginning with the Necropolis of Memphis in the north." 4.

We strongly recommend Dr. Webster's volume to the perusal of every one who is curious respecting the races that have lived and died in the Valley of the Nile.

